# TGFβ-Neurotrophin Interactions in Heart, Retina, and Brain

**DOI:** 10.3390/biom11091360

**Published:** 2021-09-14

**Authors:** Anja Schlecht, Mario Vallon, Nicole Wagner, Süleyman Ergün, Barbara M. Braunger

**Affiliations:** Institute of Anatomy and Cell Biology, Julius-Maximilians-University Wuerzburg, D-97070 Wuerzburg, Germany; anja.schlecht@uni-wuerzburg.de (A.S.); mario.vallon@uni-wuerzburg.de (M.V.); nicole.wagner@uni-wuerzburg.de (N.W.); sueleyman.erguen@uni-wuerzburg.de (S.E.)

**Keywords:** heart-brain axis, brain-retina axis, neurotrophins, TGFβ signaling, myocardial infarction, diabetic retinopathy, age-related macular degeneration, ischemic stroke

## Abstract

Ischemic insults to the heart and brain, i.e., myocardial and cerebral infarction, respectively, are amongst the leading causes of death worldwide. While there are therapeutic options to allow reperfusion of ischemic myocardial and brain tissue by reopening obstructed vessels, mitigating primary tissue damage, post-infarction inflammation and tissue remodeling can lead to secondary tissue damage. Similarly, ischemia in retinal tissue is the driving force in the progression of neovascular eye diseases such as diabetic retinopathy (DR) and age-related macular degeneration (AMD), which eventually lead to functional blindness, if left untreated. Intriguingly, the easily observable retinal blood vessels can be used as a window to the heart and brain to allow judgement of microvascular damages in diseases such as diabetes or hypertension. The complex neuronal and endocrine interactions between heart, retina and brain have also been appreciated in myocardial infarction, ischemic stroke, and retinal diseases. To describe the intimate relationship between the individual tissues, we use the terms heart-brain and brain-retina axis in this review and focus on the role of transforming growth factor β (TGFβ) and neurotrophins in regulation of these axes under physiologic and pathologic conditions. Moreover, we particularly discuss their roles in inflammation and repair following ischemic/neovascular insults. As there is evidence that TGFβ signaling has the potential to regulate expression of neurotrophins, it is tempting to speculate, and is discussed here, that cross-talk between TGFβ and neurotrophin signaling protects cells from harmful and/or damaging events in the heart, retina, and brain.

## 1. Introduction

Cardiovascular diseases such as myocardial infarction and ischemic stroke are prevailing causes of death worldwide [1,2,3]. Ischemia, a hallmark of both disorders, unavoidably leads to cell death and concomitant impaired function of the respective organ. Moreover, ischemia in the eye promotes the progression of (neo)vascular retinopathies such as diabetic retinopathy (DR) and age-related macular degeneration (AMD), which are amongst the leading causes of blindness in the western world [4,5]. Intriguingly, there is a growing body of evidence that, following (ischemic) insults, neuronal and endocrine interactions between heart and brain as well as retina and brain promote recovery of the affected tissue [6,7,8]. Collectively these interactions can be referred to as the heart-retina-brain axis. Neurotrophins and transforming growth factor β (TGFβ) family ligands might constitute regulators along this axis as they are upregulated following ischemic insults in the heart, retina, and brain [6,9,10,11,12,13,14,15,16].

**Figure 1:**
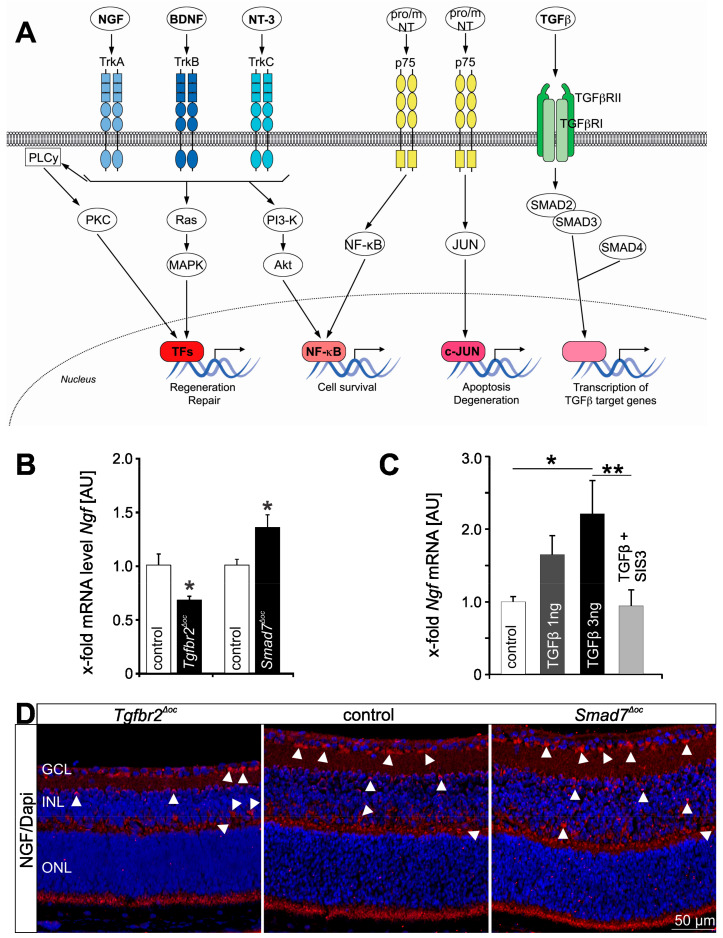
Neurotrophin and TGFβ signaling. (**A**) Simplified scheme showing neurotrophin and Smad-dependent TGFβ signaling pathways. (**B**) Quantitative real time RT-PCR (qPCR) of *Ngf* mRNA expression in *Tgfbr2^Δoc^* and *Smad7^Δoc^*-deficient and in their respective control littermate’s retinae. Data are mean ± SEM, n ≥ 5, * *p* = 0.0214 (*Tgfbr2^Δoc^*), * *p* = 0.0366 (*Smad7^Δoc^*). Expression is normalized to *Gapdh.* (**C**) QPCR analyses of RNA samples from neuronal cells (RGC-5 cells) treated with TGFβ2 (1 and 3 ng) or in combination with SIS3 (1 µM + 3 ng TGFβ2), an inhibitor of Smad3 phosphorylation. *Ngf* mRNA expression significantly increases following TGFβ treatment and its expression reverses to control levels if TGFβ is inhibited. Data are mean ± SEM, *n* ≥ 4, * *p* = 0.0119 (3 ng TGFβ2), ** *p* = 0.0059 (SIS3 + TGFβ2). Expression is normalized to *Gapdh*. (**D**) NGF immunoreactivity (red) in the retinae of *Tgfbr2^Δoc^*, control and *Smad7^Δoc^*-deficient mice at postnatal day 10. Nuclei are DAPI stained (blue). In the retina of the *Smad7^Δoc^*-deficient mouse, NGF immunoreactivity is considerably stronger with distinct preference for the perikarya (arrowheads) in the ganglion cell, inner nuclear and outer plexiform layer. In contrast labeling of the perikarya in the ganglion cell, inner nuclear and outer plexiform layer is less frequent and intense in the control retina and rarely observed in the *Tgfbr2^Δoc^* retina. The original data from (**B**–**D**), that we show in a slightly modified representation here, have been published in [42]. NGF = nerve growth factor, BDNF = brain-derived neurotrophic factor, NT-3 = neurotrophin 3, proNT = pro Neurotrophin, mNT = mature neurotrophin, TGFβ = transforming growth factor β, Trk A–C = tropomyosin receptor kinase A–C, p75 = p75 neurotrophin receptor, TGFβRI/II = transforming growth factor beta receptor 1/2, PLC*y* = phospholipase C gamma, PKC = protein kinase C, MAPK = mitogen-activated protein kinase, TFs = transcription factors, PI3-K = phosphoinositide 3-kinase, NF-kB = nuclear factor kappa-light-chain-enhancer of activated B cells, JNK = c-Jun N-terminal kinase, GCL = ganglion cell layer, INL = inner nuclear layer, ONL = outer nuclear layer.

The neurotrophin family of growth factors controls development, survival, and function of neurons in the central and peripheral nervous systems (CNS/PNS) [17,18,19,20,21]. More recently, it has been shown that neurotrophins are not only required for the nervous system, but also for development and maintenance of the (cardio)vascular system [22,23]. The mammalian neurotrophin family (Figure 1A) consists of four ligands: Nerve growth factor (NGF) [20], brain-derived growth factor (BDNF) [19], neurotrophin-3 (NT-3) [24], and neurotrophin-4/5 (NT-4/5) [25]. Neurotrophins are synthesized as precursor proteins (pro-neurotrophins) and proteolytically cleaved to their mature forms, which signal through tropomyosin receptor kinases (Trks) A–C and neurotrophin receptor p75 (p75^NTR^), although biological activity has also been ascribed to pro-neurotrophins [26,27,28,29,30]. While each of these receptor types has different binding affinities for individual neurotrophins, it is now well established that Trk activation primarily mediates cell survival, whereas p75^NTR^ signaling plays, with some exceptions, a critical role in mediating apoptosis of neuronal and non-neuronal cells (Figure 1A) [26,31].

The transforming growth factor (TGF) β family constitutes a superfamily of cytokines with a broad variety of different cellular functions such as cell development, differentiation and survival, morphogenesis and angiogenesis, tissue homeostasis, inflammation and remodeling [32,33,34,35,36,37,38,39,40,41,42]. The TGFβ superfamily includes TGFβs (TGFβ1, -β2, and -β3), bone morphogenetic proteins (BMPs), growth differentiation factors (GDFs), activins, inhibins, nodal, and anti-Mullerian hormone proteins [43,44]. TGFβ1-3 isoforms are transcribed from individual promoters and show tissue-specific expression patterns that become particularly obvious when studying the phenotypes of the different TGFβ isoform mutants [45,46]. Accordingly, mice with a deletion of TGFβ1 present defects in hematopoiesis and vasculogenesis leading to early embryonic death in half of the TGFβ1-null embryos [47]. Moreover, excessive inflammation leads to multi-organ failure after weaning in the surviving TGFβ1-deficient mice [48]. TGFβ2-deficient mice show multiple developmental abnormalities that affect the neural, visual, auditory, cardiopulmonary, urogenital and skeletal systems finally resulting in perinatal mortality [37]. However, mice lacking the TGFβ3 isoform develop a cleft palate and die immediately after birth as they cannot suckle effectively [38,49]. Intriguingly, all three isoforms are present in wound healing and repair [50], yet high amounts of TGFβ1 are released from alpha-granules of platelets (thrombocytes) that are a particularly rich source of TGFβ1 (up to 100 times more than in other cell types) [45].

TGFβ isoforms are synthesized as precursor molecules, secreted in a biologically inactive (latent) forms and converted into active TGFβs by a tissue- or injury-specific activation mechanism [51,52].

Canonical TGFβ signaling is mediated by binding of TGFβ1-3 to TGFβ receptor types 1 and 2 (TGFβR1/2), transmembrane proteins with a cytoplasmic serine/threonine kinase domain (Figure 1A). Upon TGFβ binding, TGFβR1 (activin A receptor like type 1 (ALK1), ALK5) and TGFβR2 form a heteromeric complex [53]. TGFβR1/TGFβR2 clustering results in autophosphorylation and subsequent TGFβR1-mediated phosphorylation of the intracellular effector proteins SMAD2 and SMAD3 (abbreviation refers to “small worm phenotype” (SMA) and “mother against decapentaplegic” (MAD), which are homologs of *C. elegans* and drosophila, respectively) [53]. Phosphorylated SMAD2/3 then form a complex with SMAD4 and translocate into the nucleus for the transcription of TGFβ-dependent target genes [53,54]. Quite intriguingly, it has been shown by others and our group that TGFβ signaling regulates the transcription of neurotrophins such as NGF (Figure 1B–D), BDNF and TrkB [42,55,56] and/or modulates the function of certain neurotrophins [57].

Hence, it is tempting to speculate that neurotrophins and TGFβs activate a mutual protective signaling network that interacts delicately with the ultimate goal of protecting cells from harmful and/or damaging events. We therefore address the impact of TGFβ and neurotrophin signaling in healthy and diseased heart, retina, and brain. Furthermore, we will address some of their cell type-specific effects in particular during tissue regeneration following ischemic insults or (neo)vascular retinopathies.

## 2. TGFβ and Neurotrophin Signaling in the Heart

### 2.1. Neurotrophins in the Heart

Initially, neurotrophins were intensively and exclusively studied by neuroscientists and showed tremendous and severe developmental neuronal defects in the corresponding mutant mice affecting, e.g., sensory, dorsal root, and sympathetic neurons [58,59,60,61,62,63,64]. However, besides being expressed in neuronal tissue, neurotrophins and their receptors are also expressed in cardiovascular cells such as endothelial cells, vascular smooth muscle cells, cardiomyocytes and fibroblasts as well as in immune cells such as granulocytes [65,66,67,68,69,70] (www.proteinatlas.org, accessed on 7 February 2021). Consequently, based on many thoroughly conducted studies with various neurotrophin/Trk-deficient mouse models, it became evident that neurotrophins largely exceed their role in neural regulation of heart function and are also essential regulators of cardiovascular development [65,70,71,72]. Accordingly, BDNF-deficient mice demonstrate several non-neuronal cardiac phenotypes such as defects in atrial septation and intramyocardial vessel fragility and hemorrhage, finally resulting in early postnatal lethality [71]. Moreover, ultrastructural analysis of intramyocardial arterioles and capillaries from BDNF-deficient mice shows enlarged endothelial cells with cytoplasmic vacuolization and detachment from the underlying basement membrane, indicating that BDNF promotes stabilization of cardiac arterioles and capillaries, most likely through signaling to TrkB-expressing endothelial cells and/or pericytes/vascular smooth muscle cells [71]. Accordingly, TrkB-deficient mice demonstrate a significant loss of intramyocardial blood vessels and cell type-specific deletion of TrkB in pericytes/vascular smooth muscle cells results in reduced covering of cardiac vessels, an irregular endothelial cell ultrastructure and increased vascular permeability, indicating that neurotrophin-mediated activation of TrkB signaling is essential for cardiac vascularization and critical for pericyte/smooth muscle cell development and maintenance [70,73].

### 2.2. TGFβ Signaling in the Cardiovascular System

TGFβ isoforms are constitutively expressed in cardiomyocytes, endothelial cells, and fibroblasts in both embryonic and adult mammalian hearts [74,75] (www.proteinatlas.org, accessed on 7 February 2021). Their receptors, TGFβR1 and -2, are expressed in endothelial cells, smooth muscle cells, cardiomyocytes, and fibroblasts as well as in macrophages and other leucocytes [74,75] (www.proteinatlas.org, accessed on 7 February 2021). Comparable to neurotrophins, evidence for a fundamental role of TGFβ signaling in early vascular morphogenesis arose from studying mice with deletion of different components of the TGFβ signaling pathway, such as TGFβ1 [47], TGFβR1 [76,77], TGFβR2 [40], endoglin [78,79], or SMAD5 [80]. These mutant mice develop vascular abnormalities and die during early development. Moreover, cell type-specific TGFβR1/ALK5-deficient mice revealed that TGFβR1/ALK5-mediated signaling plays a cell-autonomous role in epicardial and endocardial cells by regulating cellular communication as well as differentiation and proliferation, but is not essential in myocardial cells [81]. Furthermore, epicardial TGFβR1/ALK5-deficient mice demonstrate considerable defects of the smooth muscle cell layer surrounding the coronary arteries and aberrant formation of capillary vessels in the myocardium, indicating TGFβR1/ALK5-mediated regulation of vascular homeostasis during cardiogenesis [81]. In addition to the fundamental impact on cardiovascular developmental, TGFβ signaling is equally important for tissue maintenance in the adult heart, as cardiomyocyte-specific deletion of SMAD4 is associated with impaired ventricular function, ion-channel gene expression and cardiomyocytes survival [82].

### 2.3. A Brief Overview: Ischemic Heart Disease and Myocardial Infarction (MI)

**Figure 2:**
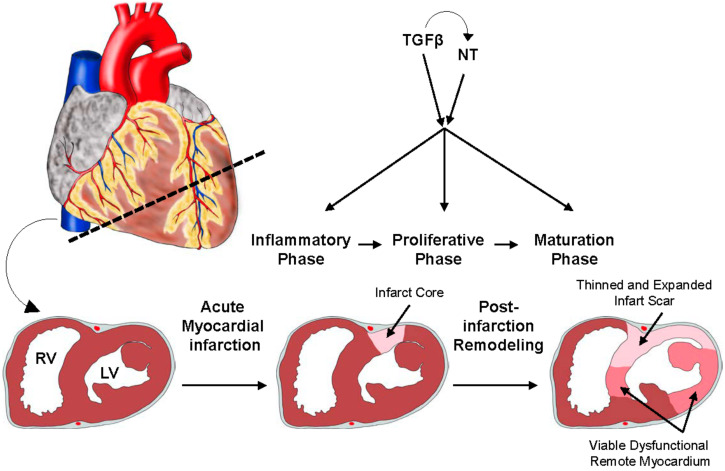
Neurotrophin and TGFβ signaling in myocardial infarction (MI). Scheme illustrating the three phases following MI that comprise the inflammatory, proliferative, and maturation phase. During each phase, multiple cellular processes occur, with the ultimate goal of healing the affected tissue. Neurotrophins and TGFβ are upregulated following MI and modulate processes in the inflammatory, proliferative, and maturation phase. TGFβ = transforming growth factor β, NT = neurotrophins.

Cardiovascular disorders including ischemic heart disease are the leading cause of death worldwide and comprise syndromes like atherosclerosis, including coronary artery disease that clinically manifests as ischemic cardiomyopathy and myocardial infarction (MI) [1,2]. MI is typically caused by formation of an atherosclerotic plaque leading to a high-grade luminal narrowing of a coronary artery that results in insufficient blood supply and thus ischemia in the cardiac tissue downstream of the affected vessel (Figure 2) [43]. Cardiomyocytes are immediately perturbed in their contractility and as early as 20–30 min after the onset of ischemia, the affected sub-endocardial cardiomyocytes demonstrate irreparable alterations, and prolonged ischemic intervals unavoidably lead to cardiomyocyte cell death [83,84]. As the regenerative capacity of the adult heart is neglectable, the remaining tissue reacts with the formation of a collagen-based, fibrotic scar [85]. This scenario involves a finely orchestrated and complex sequence of inflammatory and reparative responses occurring in three partially overlapping phases: the inflammatory, the proliferative, and the maturation phase with the ultimate goal of replacing the necrotic cardiac tissue with connective tissue (Figure 2) [85,86]. In the inflammatory phase (approx. 3–4 days (d) after MI in mice), initiated by intense sterile inflammation, pro-inflammatory cytokines are critically involved in promoting macrophage activation to clear the wound from debris and dead cells [43,87]. The proliferative phase (approx. 4–8 d in mice) is characterized by the resolution of inflammation, proliferation of (myo)fibroblasts, and neovascularization [88,89]. In the subsequent maturation phase the production of a collagen-based extracellular matrix (ECM) network finally results in the formation of a fibrotic scar [89,90,91].

Macrophages are critically involved in regulation of inflammation and scar formation following MI. During the course of MI they change their transcriptional polarization from (1) pro-inflammatory, ECM-degrading on day 1 to (2) proliferative, phagocytic on day 3 to (3) pro-reparative, ECM remodeling, scar formation on day 7 post MI [87].

Intriguingly, as components of both TGFβ and neurotrophin signaling, have been shown to be upregulated following MI, both pathways are considered important players in the regulation of the inflammatory, proliferative, and maturation phase [6,9]. Experimentally induced cardiac ischemia through ligation and reperfusion of the left anterior descending coronary artery results in a significant up-regulation of *Tgfb1–3* mRNA in the reperfused infarct area in mice and pigs [9,10]. In non-reperfused MI increased expression of *Tgfb1–3* mRNA concomitant with SMAD2/3 phosphorylation in interstitial cells and cardiomyocytes in the border zone [10,92,93] as well as increased long-term (8 weeks post MI) expression of TGFβ1, TGFβR2, SMAD2, SMAD3 and SMAD4 in the border and fibrotic zones are observed [94]. Similarly, TrkB expression is increased in particular in cardiomyocytes in the border zone following non-reperfused, experimentally induced MI concomitant with marked increase in plasma BDNF levels and upregulation of BDNF expression in the brain [6]. Moreover, following injury to the abdominal and thoracic aorta using a balloon embolectomy catheter, BDNF and TrkB expression are induced in neointimal vascular smooth muscle cells [66].

The observed correlation between increased expression of TGFβ and neurotrophin signaling components and experimentally induced MI suggests important contributions of both pathways to the delicately orchestrated sequence of inflammatory and reparative responses following MI. This was further elucidated using knockout mouse models of these signaling pathways as discussed below. Of note, data from knockout animal/MI studies that we address in this review originate either from animals of both sexes or the specific sex was not mentioned. Thus, we cannot comment on potential gender-specific effects of neurotrophin/TGFβ signaling in MI [95].

### 2.4. Neurotrophins in MI

Mice with induced systemic deletion of BDNF (CAG-Cre-ER; *Bdnf^flox/flox^*) show a significantly increased number of apoptotic cardiomyocytes 10 d post MI (non-reperfused) concomitant with dysregulated pro- and anti-apoptotic factors such as BAX (BCL2 Associated X, Apoptosis Regulator) and BCL2 (BCL2 Apoptosis Regulator), impaired vascularization of the border zone, and a larger fibrotic area two weeks post-MI [6]. Other studies indicate fundamental roles of the neurotrophin BDNF in communication with immune cells and regulation of immune cell polarization during MI. For example, heterozygous BDNF knockout mice display reduced neutrophil invasion as well as angiogenesis following MI [96]. In macrophages BDNF promotes the shift from pro-inflammatory to reparative polarization, thereby modifying the inflammatory microenvironment [97]. It has recently been reported that a polymorphism of *BDNF* (Val66Met), well known to be related to mood disorders and neurodegenerative diseases, also has an impact on the reactive state of macrophages following MI, predominantly by promoting the pro-inflammatory state, thereby predisposing to adverse cardiac remodeling [98,99]. Thus, neurotrophins such as BDNF influence all three regenerative phases following MI. They have direct protective function on cardiomyocytes and contribute to the inflammatory phase through crosstalk with immune cells. Moreover, BDNF stimulates the formation of new vessels either through direct binding on TrkB on endothelial cells or by increasing vascular endothelial growth factor (VEGF) [96,100], thereby promoting vascularization during the proliferative phase of MI. In addition, fibrotic scar formation following MI is attenuated by BDNF.

### 2.5. Cell Type-Specific Effects of Neurotrophins and the Heart-Brain Axis

Cell type-specific knockout mouse models of the neurotrophin signaling pathway have been instrumental in shedding light on the role of neurotrophin signaling in heart development and function. Specific ablation of *TrkB* in cardiomyocytes (αMHC-MerCreMer; *TrkB*^flox/flox^) does not affect heart size or systolic function under physiologic conditions, a finding that might well be attributed to the fact that in the adult, healthy heart, *TrkB* is undetectable [6]. However, following MI (non-reperfused), TrkB is significantly upregulated particularly in the border zone and consequently, cardiomyocyte-specific deletion of *TrkB* results in more apoptotic cardiomyocytes, a slightly larger left ventricular diastolic dimension, a significantly decreased systolic function and a larger fibrotic area than in control littermates [6]. These data indicate a major role of TrkB-mediated neurotrophin signaling in cardiomyocytes after MI, directly affecting their survival and indirectly regulating vascularization and fibrosis [6].

Under physiological conditions, cardiomyocyte-specific, tamoxifen-inducible BDNF knockout mice (αMHC-MerCreMer; *Bdnf^flox/flox^* mice) have normal heart size and systolic function and even following MI (non-reperfused), cardiac remodeling, vascularization of the border zone and systolic dysfunction is not altered compared to wildtype controls [6]. Surprisingly, *Bdnf* is markedly downregulated in cardiac tissue after MI in both wildtype and cardiomyocyte-specific BDNF knockout mice [6]. Yet, BDNF expression significantly increased in the forebrain including thalamus and hypothalamus, resulting in significantly increased BDNF plasma levels in cardiomyocyte-specific BDNF knockout and wildtype mice following MI [6]. This finding indicates that, e.g., cardiac afferent nerve fibers transmit mechano- and chemo-sensitive information such as ischemia to the brain, which reacts by increased production of BDNF, most likely to protect the heart against ischemic injury [6].

This intimate crosstalk between brain and heart, the brain-heart axis, has been demonstrated in a series of very elegant experiments showing that systemic deletion of BDNF (CAG-Cre-ER; *Bdnf^flox/flox^* mice), deletion of BDNF in neuronal cells (Nestin-Cre; *Bdnf^flox/flox^* mice) as well as ablation of afferent sensory neurons two weeks before MI using subepicardial injections of capsaicin (CAP), inhibited plasma and brain BDNF increase following MI. Moreover, CAP-treated and Nestin-Cre; *Bdnf*^flox/flox^ mice displayed significantly impaired cardiac function and a larger fibrotic area following MI, which could be rescued by intraperitoneal injections of BDNF, clearly indicating that upregulation of BDNF in the brain is induced by cardiac afferent fibers to protect the heart against ischemic injury [6].

The protective function of BDNF is further underlined by reports from human patients with low circulating plasma BDNF levels, which is considered a critical biomarker for heart failure and has been shown to be associated with future coronary events, increased mortality in angina pectoris, and adverse left ventricular cardiac remodeling [23,98,101,102,103,104].

Taken together, neurotrophins such as BDNF clearly play a protective role in the heart after MI by regulating the inflammatory, proliferative, and maturation phase of cardiac fibrosis. Moreover, as low plasma BDNF levels can be detected before the onset of severe cardiac diseases, it could be used as a biomarker in routine examinations of patients with cardiovascular risks factors.

### 2.6. The Role of TGFβ Signaling in MI: Timing Matters

There are a number of studies addressing the role of a systemic inhibition TGF-β signaling in the context of MI showing both detrimental and protective effects.

Systemic inhibition of TGFβ signaling by transfecting the limb skeletal muscle with a plasmid encoding the extracellular domain of TGFβR2 shows different effects, depending on timing: Inhibition of TGFβ signaling seven days before MI (through permanent left coronary artery ligation) results in a dramatically enhanced mortality rate accompanied by exacerbated left ventricular (LV) dysfunction, enhanced cytokine expression and infiltration of neutrophils in mice [105]. However, when TGF-β signaling is inhibited on the day of and seven days after MI, respectively, survival rate and infarct size is not affected but LV hypertrophy and interstitial fibrosis is attenuated (Ikeuchi et al., 2004).

Accordingly, inhibition of TGFβ signaling in mice by intraperitoneal injection of an antibody that specifically neutralizes TGFβ1–3, 7 d before or 5 d after MI (non-reperfused) results in reduced type I collagen mRNA expression in the infarcted region in the early phase of MI, worsened left ventricular remodeling and increased mortality [106]. Yet, the amounts of infiltrating neutrophils were not altered in this study [106].

Systemic deletion of SMAD3 does not influence morphology or function under physiological conditions, however, following MI and reperfusion (1 h occlusion, reperfusion for 6 h to 72 h), SMAD3^−/−^ mice demonstrate a short-term (6 h/24 h) reduction of chemokine expression, reduced neutrophil invasion (24 h and 72 h post MI) and reduced fibrotic remodeling of the infarcted heart compared to MI-wildtype mice [107].

Inhibition of TGFβ signaling by an orally administered small-molecule inhibitor of TGFβR1 (GW788388) starting on the day of experimentally induced MI (non-reperfusion) in rats results in attenuated systolic dysfunction and left ventricular remodeling concomitant with a reduced number of α-smooth muscle actin-positive cells in the cardiac interstitium and reduced collagen I expression in the non-infarct zone [108]. However, in this model the number of macrophages is not altered between infarcted control and TGFβR1 inhibitor-treated rats [108].

Taken together, when systemic TGFβ inhibition is initiated before or at the onset of myocardial ischemia, detrimental effects are observed, most likely due to TGFβ signaling-mediated suppression of inflammation, activation of fibroblasts, and generation of extracellular matrix. As a side note, to our knowledge the angiogenic activity of TGFβ signaling and its potential impact on vascularization of the infarct border zone has not been addressed yet.

In contrast, systemic inhibition of TGFβ signaling three days after MI (induced by left coronary artery ligation) using adenoviral-driven expression of soluble TGFβR2 (Ad.CAG-sTbetaRII) shows a significantly higher (96%) survival rate of TGFβ signaling-attenuated mice compared to controls (71%) [109]. TGFβ-inhibited mice display less apoptotic myofibroblasts in the granulation tissue 10 days after MI as well as reduced fibrosis and improved cardiac function four weeks after MI [109]. Inhibition of TGFβ signaling at a later time point (four weeks after MI) has no effect [109].

To our understanding, the described differential effects of TGFβ signaling inhibition can be explained by (1) the different methodologies used, most likely resulting in different degrees of inhibition, and (2) the different timing of TGFβ signaling inhibition. Systemic inhibition of TGFβ signaling affects all cells involved in cardiac repair. Thus, depending on the time point of attenuation, different active cellular populations are affected. Given the complex biology of TGFβ signaling and its manifold, context- and cell type-specific functions, studies using inducible and cell type-specific deletion of TGFβ signaling have helped to further elucidate its detailed role following MI.

### 2.7. Specific Actions of TGFβ Signaling in MI: Cell-Type Matters

Mice with specific deletion of *Tgfbr1* or *Tgfbr2* in cardiomyocytes (αMHC-MerCreMer; *Tgfbr1^flox/flox^* or αMHC-MerCreMer; *Tgfbr2^flox/flox^* mice, tamoxifen treatment: 2-4 weeks before MI) are protected against MI-dependent, early onset mortality due to wall rupture and show a marked decrease in neutrophil recruitment and accompanying metalloproteinase-9 activation after MI (non-reperfused) [92]. Systemic inhibition of TGFβ signaling by administering a neutralizing antibody against TGFβ 1–3 (clone 1D11, Genzyme) starting on day one of experimentally induced MI (and 3 x/week thereafter) confirmed the detrimental effects of systemic TGFβ inhibition as all anti-TGFβ-treated animals died within 5 days, whereas control antibody-treated mice showed the expected 40% mortality rate [92]. The authors concluded that protective effects of TGFβ inhibition are most likely attributed (in part) to the function of TGFβ signaling in cardiomyocytes [92], which alters expression of molecular factors known to play a role in cardiac remodeling [92,110].

Chen and coworkers addressed the inflammatory aspects of TGFβ signaling by inhibiting its intracellular effector, SMAD3, specifically in monocytes/macrophages [93]. Myeloid cell-specific SMAD3 knockout mice (LyzM-Cre; *Smad3^flox/flox^*) present a normal baseline heart rate, regular cardiac morphology and function, but show a modest prolongation of the time from the beginning atrial depolarization to the beginning of ventricular depolarization (PR interval) under physiological conditions [93]. However, following (non-reperfused) MI, LyzM-Cre; *Smad3^flox/flox^* mice display increased late onset, post-infarction mortality and accentuated dilative post-myocardial infarction cardiac remodeling [93]. Intriguingly, despite an increased macrophage density in infarcted LyzM-Cre; *Smad3^flox/flox^* mice, they show a disturbed clearance of dead cardiomyocytes and neutrophils [93]. In vitro studies of SMAD3^−/−^ macrophages exhibited impaired phagocytic activity and higher expression of pro-inflammatory cytokines such as *interleukin (Il) 1β, TNF-α,* and *Ccl2* as well as reduced expression of anti-inflammatory mediators such as *Il-10* and *TGFβ1* [93]. Macrophages are involved in regulation of inflammation and scar formation and change their polarization from pro-inflammatory to pro-reparative during the first week following MI [87]. Their shift from inflammatory to anti-inflammatory/reparative seems to be promoted by TGFβ signaling and protects the heart from adverse post MI remodeling [93,111].

In summary, neurotrophin and TGFβ signaling play fundamental roles in inflammation and repair following MI (Figure 2). Given the fact that TGFβ signaling has the potential to regulate neurotrophins (Figure 1B–D) [42,55], it is tempting to speculate that these signaling pathways cooperate with the overall aim of protecting the tissue from harmful events such as MI. Indeed, BDNF plays a beneficial role in the heart by directly mediating protective effects on cardiomyocytes, contributing to the inflammatory phase following MI through crosstalk with immune cells and promoting a reparative phenotype in macrophages, which stimulates vascularization and reduces fibrotic scar formation following MI [6,96,97,98,100]. Yet, the role of TGFβ signaling in post MI survival and repair is more complex and clearly dependent on the time point of its inhibition and the affected cell type. Recently published studies have demonstrated that TGFβ signaling is beneficial for post MI survival by suppressing inflammation and activating fibroblasts and extracellular matrix production. However, these effects are time-dependent, as differential outcomes were reported depending on whether TGFβ signaling has been deleted before or after experimentally induced MI.

Moreover, recently published data show that TGFβ signaling in cardiomyocytes is detrimental for post MI survival [92]. In contrast, TGFβ signaling in macrophages has beneficial effects by promoting the phenotypic shift from pro-inflammatory to reparative, thereby protecting the heart from adverse remodeling resulting in enhanced survival post MI. Consequently, thinking towards therapeutic translation of TGFβ signaling modulation, further studies addressing its cell type-specific effects following MI are urgently needed.

## 3. TGFβ and Neurotrophin Signaling in the Central Nervous System (CNS): Retina

The retina develops from the optic vesicle that extends from the forebrain and is as such part of the central nervous system (CNS) [112]. Therefore, retinal tissue shares considerable similarities with the brain, such as the lack of spontaneous neuronal regeneration after damage as well as the blood-retinal barrier (BRB) protecting retinal neurons from harmful, blood-borne substances analogous to the protection of brain neurons by the blood-brain barrier (BBB) [113,114,115]. Moreover, microvascular abnormalities caused by systemic diseases such as diabetes or hypertension can be easily observed in retinal blood vessels, which allows predictions of the vascular status of other organs, e.g., the heart and/or brain. In addition to the common embryological origin of retina and brain [112], there is a close molecular relationship and interplay between retina and brain mediated by, among others, neurotrophins as discussed in the following.

### 3.1. Neurotrophins in the Retina and the Brain-Retina Axis

Comparable to the brain, neurotrophins such as BDNF, NGF, NT-3 and NT4/5 play a significant role in retinal neuronal differentiation and protection from cell death during development and in adulthood [42,116,117,118,119,120,121,122]. Neurotrophins are expressed in several retinal cell types, including Müller cells, microglia, photoreceptors, bipolar cells, the retinal pigment epithelium (RPE), and retinal ganglion cells (RGC), with BDNF and NGF expression being the best described [31,123,124,125,126] (www.proteinatlas.org, accessed on 7 February 2021). Moreover, BDNF is produced by neurons in the superior colliculus/lateral geniculate nucleus and transported retrogradely, through retinal ganglion cell axons, to retinal ganglion cell bodies [127,128]. Intriguingly, interruption of this retrograde transport led to the “neurotrophin deprivation theory” that is discussed as a possible scenario for retinal ganglion cell death in glaucoma, a complex multifactorial ocular disease that is amongst the leading causes of blindness worldwide [7,129,130]. TrkB is mainly expressed in retinal ganglion, horizontal, amacrine, and Müller cells and to a lower extent in photoreceptor cells (www.proteinatlas.org, accessed on 7 February 2021, [131,132,133,134]). Upon BDNF-TrkB binding, signaling pathways such as the phosphatidylinositol 3-kinase (PI3-K)-Akt pathway are activated resulting in, e.g., inhibition of pro-apoptotic targets, as well as induction of various transcription factors promoting neuronal survival [135,136,137,138]. To date, the neuroprotective role of BDNF, in particular for retinal ganglion cell survival, has most extensively been studied [139,140]. In BDNF-deficient mice, the functional development of RGCs and the transmission of photoreceptor signals to bipolar cells are impaired [131,141,142]. Furthermore, the size of the optic nerve in these mice is clearly reduced [143]. TrkA and p75^NTR^ are expressed in RGCs, photoreceptors, and Müller cells suggesting that these cells are responsive to (pro)NGF [31]. NT-3 mediates its function by binding to TrkC, a receptor mainly expressed in photoreceptors and Müller cells, while BDNF and NT-4/5 preferentially bind to TrkB [144]. As briefly mentioned previously, neurotrophin signaling through TrkA-C mediates neuronal survival, whereas p75^NTR^ signaling is, with some exceptions, involved in mediating apoptosis [31,145,146,147,148,149,150,151]. Taken together, neurotrophins contribute to the close relationship between the brain and retina, which could be defined as the brain-retina axis. Disturbances within this axis impact the pathogenesis of ocular diseases such as glaucoma [7]. Intriguingly, a comparable relationship between the brain, ear, and heart, the brain-ear-heart axis, is implicated in neurodegenerative Alzheimer’s disease [152]. However, while the protective effects of neurotrophins in the context of retinal neurodegeneration have been studied extensively in recent years [139,140,146,153,154,155,156,157], there is also emerging evidence that neurotrophins may contribute to the pathogenesis of vascular pathologies in the retina.

### 3.2. TGF-β Signaling and Its Cross-Talk with Neurotrophins in the Eye

**Figure 3:**
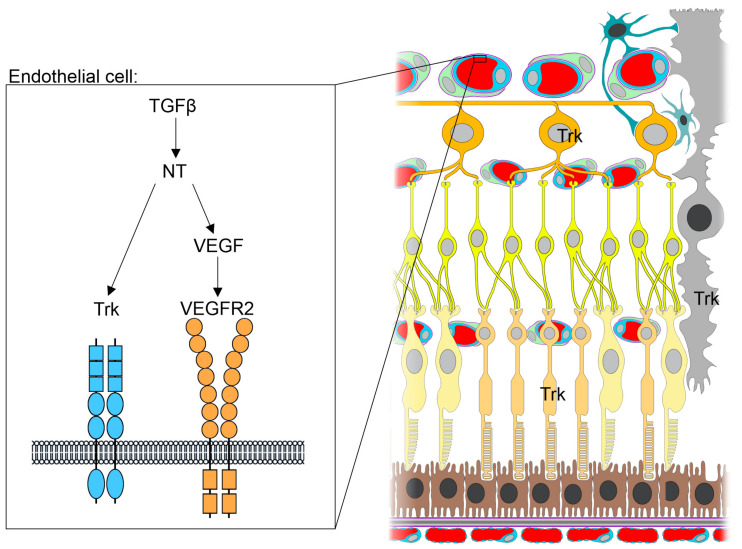
Angiogenic properties of neurotrophin and TGFβ signaling. Scheme showing the interplay of neurotrophins, TGFβ signaling, and endothelial cells in the retina. TGFβ mediates upregulation of neurotrophins in the retina, which promote angiogenesis either by direct binding to Trk receptors on endothelial cells, or by inducing VEGF expression and subsequent activation of VEGFR2. TGFβ = transforming growth factor β, VEGF = vascular endothelial growth factor, VEGFR2 = vascular endothelial growth factor receptor 2, Trk = tropomyosin receptor kinase.

All three TGFβ isoforms can be detected in the aqueous and vitreous humor of the mammalian eye, however their expression levels vary, with TGFβ 2 being the dominant isoform under physiological conditions and TGFβ 1 only being detectable in its latent from [149,158,159]. In the anterior segment of the eye, immunohistochemical staining shows that TGFβ 1 is present in the limbal epithelium, ciliary processes, and blood vessels of the ciliary body, whereas TGFβ 2 is detected additionally in the conjunctiva [160]. Both, corneal epithelial and endothelial cells express *Tgfb-3* mRNA [161]. In the posterior segment of the eye, all three TGFβ isoforms are expressed in retinal neurons, microglial cells, hyalocytes, endothelial cells and pericytes (www.proteinatlas.org, accessed 7 February 2021) [162,163]. Moreover, TGFβ2 and -3 are expressed in vascular smooth muscle cells and in the choroid [162,163]. In the RPE, as in the aqueous humor and vitreous, TGFβ2 is the predominant isoform and its expression in the RPE is several-fold higher compared to the neurosensory retina [164,165,166]. In the retina, TGFβR1 and -2 are expressed in neurons and Müller cells, vascular cells, and the retinal pigment epithelium [167,168] (www.proteinatlas.org, accessed 7 February 2021). Canonical TGFβ signaling plays essential roles in cell growth, migration, proliferation and cell death, both during ocular development as well as in tissue homeostasis during adulthood [34,37,42,169,170,171,172,173,174,175,176]. TGFβ2-deficient mice exhibit abnormal ocular morphogenesis with a thin corneal stroma, absence of corneal endothelium, and fusion of the cornea with the lens [37,176]. Our group recently reported that embryonic deletion of TGFβR2 in retinal neurons and Müller cells results in increased apoptosis of retinal neurons during ontogenesis and in impaired retinal function [42]. Intriguingly, we could reverse this effect by increasing TGFβ activity by specific deletion of SMAD7 in retinal neurons and Müller cells, resulting in a disinhibition of TGFβ signaling [42]. We could furthermore demonstrate that TGFβ2 treatment of retinal neuronal cells results in increased expression of NGF (Figure 1C) [42]. Accordingly, NGF expression is reduced in TGFβR2-deficient animals and significantly enhanced in animals with disinhibition of TGFβ signaling (Figure 1B,D), indicating that TGFβ indeed has the ability to regulate transcription of neurotrophins such as NGF (Figure 3) [42].

Inhibition of TGFβ1 and -3 in adult animals by systemic expression of soluble endoglin results in apoptosis of neuronal and vascular cells in the retina, concomitant with impaired retinal function [175]. Moreover, systemic inhibition of TGFβs results in reduced perfusion of retinal vessels and loss of capillary integrity [175].

TGFβ signaling is critical for vasculo-/angiogenesis during early embryonic development and it is similarly important for vascularization of the CNS [47,177,178,179]. Our group recently showed that deletion of TGFβ signaling in neonatal mice using tamoxifen-induced deletion of *Tgfbr2* (CAGGCre-ER; *Tgfbr2^flox/flox^*) in the entire eye results in impaired retinal vascularization with displaced retinal vessels, abundant microaneurysms, leaky capillaries, and retinal hemorrhages [41]. We furthermore showed that retinal capillaries have a thickened basal lamina and are not covered by differentiated pericytes, but by vascular smooth muscle-like cells [41]. Intriguingly, specific deletion of *Tgfbr2* in endothelial cells results in multiple micro-haemorrhages and glomeruloid vascular tufts in the retina and throughout the cerebral cortex and hypothalamus [35,177]. Furthermore, in these mutant mice the deeper retinal vascular plexus failed to form and the angiogenic sprouts stalled in the inner nuclear layer [177]. These studies suggest, that TGFβ signaling in vascular endothelial cells is pivotal for maintaining vascular integrity and migration into developing neural tissues in early postnatal life.

In summary, TGFβ signaling not only mediates neuroprotection, but it is equally important in vascular development and maintaining vascular integrity. Yet again, the role of TGFβ signaling is complex and context- and cell type-dependent, as elevated TGFβ levels are considered to contribute to extracellular matrix reorganization in ocular diseases such as primary open angle glaucoma [180,181].

### 3.3. A Brief Overview: (Neo)Vascular Eeye Diseases

Age-related macular degeneration (AMD) is a common ocular disease accounting for 5% of all blind adults (≥50 years) [4]. In the elderly population it is among the most common causes of blindness worldwide [4]. As the disease progresses, two stages of AMD can be differentiated: the dry form, also known as geographic atrophy and the wet stage, also known as neovascular AMD (nAMD) [182,183,184]. Geographic atrophy leads to progressive, but rather slow vision loss, caused by deterioration of the retinal pigment epithelium and photoreceptors [185]. As the disease progresses, local hypoxia initiates the transition of the disease from the dry to the wet form (nAMD) [186,187,188]. The main hallmark of nAMD is the development of choroidal neovascularizations (CNV) leading to rapid and severe vision loss in patients suffering from this disease [182,183,184]. CNV are defined by the formation of new blood vessels originating from the choroidal vasculature and penetrating the Bruch membrane and subretinal space concomitant with edema formation and hemorrhage [189]. In the context of CNV formation, vascular endothelial growth factor (VEGF) is considered a master regulator, since high concentrations of VEGF-A, a potent pro-angiogenic factor, have been measured in the aqueous humor of nAMD patients [190]. In recent years, anti-VEGF therapy has enabled significant advances in the treatment of neovascular AMD, but treatment success is hampered by the short intraocular half-life of antibodies and requires frequently repeated intraocular injections [191]. Furthermore, one third of patients with neovascular AMD (nAMD) loses vision despite continuous anti-VEGF therapy [191], indicating other disease-associated molecular mediators. To mimic disease progression of nAMD as closely as possible in animal models, the laser-induced CNV model has been developed and used as the gold standard for several years [192,193]. In this model, the Bruch membrane and the RPE are punctually disrupted via laser application leading to the induction of CNV accompanied by inflammation, similar to what is observed in human patients with nAMD [194].

Diabetic retinopathy (DR) is the leading cause of blindness in the working population and, similar to AMD, is associated with pathological neovascularization during disease progression [5]. DR is mostly asymptomatic during early stages, however, first clinical signs of non-proliferative DR include microaneurysms and small hemorrhages [195]. Furthermore, occlusion of retinal vessels can be observed, leading to development of ischemic infarcts of the nerve fiber layer, also known as cotton wool spots [196]. As the disease progresses, ischemia caused by non-perfused capillaries leads to upregulation of pro-angiogenic factors, such as VEGF, resulting in increased vascular permeability, driving retinal and vitreal neovascularization, a scenario clinically named as proliferative DR (PDR) [196,197,198,199]. These neovascularizations are fragile and mostly consist of fenestrated endothelium, thus being more susceptible to leakage and hemorrhage [196,199]. Repeated vitreous hemorrhages in the late phase of the disease lead to the formation of fibrous, tractive vitreal membranes, resulting in retinal detachment and thus pronounced vision loss [196,199].

### 3.4. Neurotrophins in (Neo)Vascular Eye Diseases

Besides the well-known role of neurotrophins in mediating growth, differentiation, and survival of neuronal and non-neuronal cells [31,42,116,117,118,119,120,122,138,200], there is emerging evidence that they contribute to the development of neovascularization in diseases such as diabetic retinopathy (DR) or age-related macular degeneration (AMD), too [11,201,202]. Neurotrophins mediate their pro-angiogenic effect either directly by binding to Trk receptors on endothelial cells or indirectly by inducing expression of VEGF in other cells (Figure 3) [12,96,100,203,204,205]. Accordingly, inhibition of VEGF via intravitreal application of bevacizumab, frequently used to inhibit neovascularization in patients suffering from (n)AMD or DR, results in a significant decrease of NGF levels in the retina [206]. Moreover, NGF treatment of choroidal endothelial cells increases their migration by 50%, indicating that neurotrophins have the capacity to modulate neoangiogenic processes as seen in DR or AMD [201,207]. Furthermore, immunohistochemical staining shows increased retinal NGF expression in a rat model of AMD-like retinopathy [11] and NT-3 and NT4/5 levels are elevated in vitreous samples of animals with experimentally induced proliferative DR [12].

However, there are conflicting reports on neurotrophin expression/levels in humans suffering from AMD or DR. Intriguingly, BDNF is significantly increased in the serum of patients with dry and wet AMD, but BDNF levels are decreased in the aqueous humor of AMD patients [202,208]. There are studies reporting significantly increased neurotrophin levels (BDNF, NGF, NT3 and NT4/5) in the vitreous of DR patients [12,209], but others demonstrate significantly decreased BDNF and NGF levels in serum and aqueous humor of DR patients [8,210]. Comparable to patients with cardiovascular diseases, decreased serum BDNF and NGF levels can be detected before the onset of DR and are therefore discussed as biomarkers and critical indicators of DR development.

### 3.5. TGF-β Signaling in (Neo)Vascular Eye Diseases

**Figure 4:**
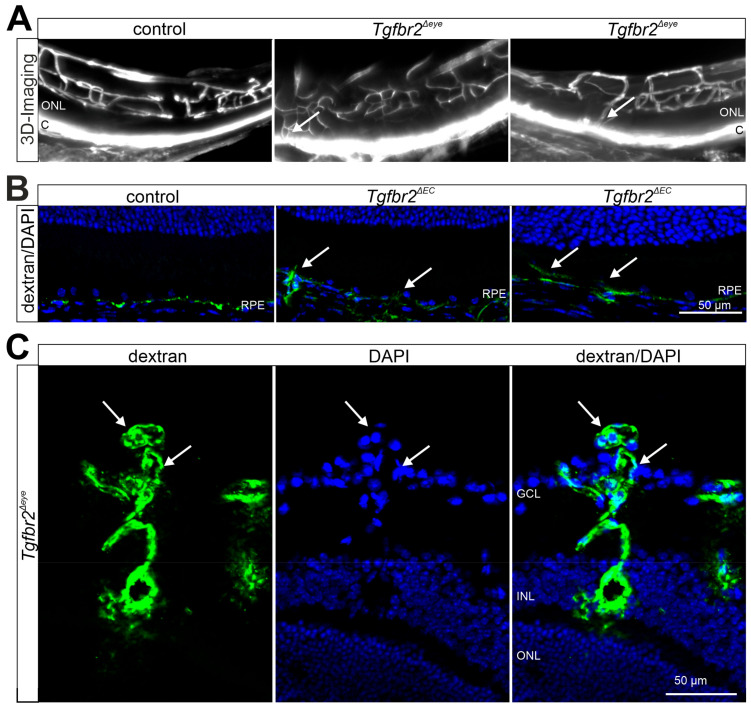
Deletion of TGFβ signaling results in retinal and choroidal neovascularization. (**A**) Light-sheet fluorescence microscopy of cleared eyes of 6-week-old lectin-injected Tgfbr2^Δeye^ mice and a control littermate. The control mouse shows an essentially regular arborized retinal vasculature. The Tgfbr2^Δeye^ mice have an irregular arrangement of the retinal plexus and develop choroidal neovascularization forming anastomoses between retinal and choroidal vessels (arrows). (**B**) FITC-dextran (green) perfused retinal meridional sections of 6-week-old *Tgfbr2^ΔEC^* mice and a control littermate. White arrows point toward tracer leakage in the retinal pigment epithelium (RPE; middle panel) and choroidal neovascularization (right panel) invading the RPE and subretinal space. Nuclei are DAPI stained (blue). (**C**) Meridional section of a 6-week-old FITC-dextran (green) perfused *Tgfbr2^Δeye^* retina shows neovascularization into the vitreous (white arrows), similar to what can be observed in patients suffering from proliferative diabetic retinopathy. Nuclei are DAPI stained (blue). The original data which we show in a slightly modified representation here, have been published in [35,41]. GCL = ganglion cell layer, INL = inner nuclear layer, ONL = outer nuclear layer, RPE = retinal pigment epithelium.

In this context, TGFβ signaling is controversially discussed as a potential regulator of nAMD, as both inhibition as well as induction of this pathway has been linked to the disease [211]. Using the laser-induced CNV model, several studies show an upregulation of TGFβ1 and -2 after laser CNV induction [212,213,214], supporting the proangiogenic role of TGFβ signaling in nAMD. Furthermore, Wang and colleagues found that inhibition of TGFβ signaling via application of inhibitors of TGFβ1 or TGFβR1 in mice results in both reduced levels of VEGF-A and decreased CNV severity after laser treatment [214]. Comparable results are also obtained in the rat model of laser-induced CNV, in which a reduction of CNV formation is observed after systemic application of TGFβ inhibitory peptides in both the early and late phases of CNV development [215,216]. However, not only results from animal models emphasize the proangiogenic properties of TGFβ signaling in the context of CNV development. Analysis of patients with nAMD confirmed elevated TGFβ1 levels in vitreous as well as aqueous humor [217]. Furthermore, increased TGFβ1 and -2 expression could also be detected in the RPE of nAMD patients [212,218,219]. As mentioned above, there is also evidence that TGFβ signaling is anti-angiogenic and consequently hampers CNV development. In vitro data show that TGFβ2 expression by Müller glial cells has a direct inhibitory effect on the proliferation behavior of retinal endothelial cells [220]. Accordingly, our group recently showed that inhibition of TGF-β signaling in (1) the entire eye (CAG-Cre-ER;*Tgfbr2^flox/flox^*) (Figure 4A) or (2) vascular endothelial cells (Figure 4B) (Cdh5-cre/ERT2;*Tgfbr2^flox/flox^*), specifically, using Cre-loxP mediated, inducible deletion of TGFβR2 in mice, leads to CNV formation [35]. Moreover, following inhibition of TGF-β signaling and subsequent CNV development, we observed activation of the local immune system, comparable to human patients suffering from nAMD [35,221,222]. There are also reports suggesting an anti-angiogenic effect of TGFβ signaling in human patients with nAMD. Tosi and colleagues show significantly decreased levels of TGFβ2 in aqueous humor samples after nAMD onset [217]. As TGFβ2 is the predominant isoform in ocular tissues, this finding is of great relevance as it might indicate protective effects of TGFβ signaling from CNV formation.

There is also evidence that TGFβ signaling may be involved in development and progression of DR, too. Animal experiments with diabetic rats revealed increased expression of ALK5 (TGFβR1) as well as increased levels of TGFβ1 and phospho-Smad2 in retinal vessels compared to control rats [223]. These data are consistent with clinical studies showing increased levels of TGF-β in the vitreous of patients with proliferative DR [224]. Furthermore, polymorphisms in the *Tgfb1* gene, such as the R25P polymorphism, are being discussed as potential risk factors for DR as *Tgfb1* R25P leads to increased expression of TGFβ and is associated with increased risk of DR [225]. In contrast, a study by Spranger and colleagues demonstrates a lower concentration of TGFβ in the vitreous of patients with proliferative DR compared to control patients, although different TGFβ isoforms were not discriminated [226]. This finding might be explained by the progressive thickening of the basement membrane in DR, which prevents or impedes direct contact between endothelial cells and pericytes required for transition of latent TGFβ to its active form [227,228,229]. Our group recently showed that deletion of the TGFβ signaling pathway in all ocular cells of newborn mice induced a phenotype that reflects the characteristics of non-proliferative as well as proliferative DR with numerous retinal microaneurysms and neovascularization, leaky capillaries, and associated hemorrhages (Figure 4C) [41]. Furthermore, these mice showed a reduced number of differentiated pericytes and a thickened basement membrane [41]. Over time, non-perfused ghost vessels developed, accompanied by local hypoxia and induction of pro-angiogenic factors such as VEGF-A, which in turn lead to the formation of retinal and vitreal neovascularizations [41]. In late-stage pathology, retinal detachment caused by tractive membranes was observed, concomitant with neuronal apoptosis and reduced neuronal function [41].

In summary, the current state of research indicates a fundamental role of the TGF-β signaling pathway in the development of pathologic vascular changes in the eye, although the exact molecular mechanisms have not yet been elucidated. Moreover, as both TGFβ and neurotrophin signaling play a significant role in neuroprotection and development of neovascular ocular diseases, it is tempting to speculate as to whether and how these pathways interact. Indeed, there is evidence supporting the concept of direct interplay between TGFβ and neurotrophin signaling in the retina. Our group recently showed that mice with deletion of TGFβ signaling in retinal neurons and Müller cells display significantly more apoptosis during retinal development compared to control animals [42]. Moreover, mice with deletion of the inhibitory SMAD7 gene display enhanced activation of TGFβ signaling in retinal neurons and Müller cells and less apoptosis during retinal development [42]. In this study we could furthermore demonstrate that TGFβ2 treatment of retinal neuronal cells results in increased expression of NGF concomitant with decreased apoptosis (Figure 1C) [42]. Accordingly, NGF expression was reduced in *Tgfbr2*-deficient mice and significantly enhanced in animals with disinhibition of TGFβ signaling (Figure 1B,D) [42]. Another study by Ma and colleagues describes how BDNF expression in retinal microglial cells is significantly reduced upon inhibition of TGFβ signaling in microglia using inducible knockout mice (Cx3cr1-Cre-ER; *Tgfbr2^flox/flox^*) [56]. Thus, TGFβ signaling indeed has the ability to regulate transcription of neurotrophins such as NGF and BDNF, through which it might mediate, at least in part, its neuroprotective and/or angiogenic function.

## 4. TGFβ and Neurotrophin Signaling in the Central Nervous System (CNS): Brain

### 4.1. Neurotrophins in Nervous System Development and Homeostasis

In the brain, neurotrophin receptors are widely expressed by neurons, and both neurons and glial cells produce biologically active neurotrophin ligands [230]. Astrocytes, in addition to their metabolic support of neurons, also secrete neurotrophins and are promising therapeutic targets for neuroprotection and neuroregeneration in ischemic stroke [231]. Different neurotrophins regulate survival and plasticity of different neuronal subsets during development as well as in the adult organism. For example, deletion of NGF or its receptor TrkA in mice results in perinatal loss of sensory and sympathetic neurons, yet basal forebrain cholinergic neurons develop normally [58,59]. In contrast, NGF inhibition in adult transgenic mice results in severe deficits in basal forebrain cholinergic neurons [232]. Mutant mice lacking BDNF have severe deficiencies in coordination and balance, associated with excessive degeneration in several sensory ganglia including the vestibular ganglion [233]. NT3 loss-of-function mutant mice display severe movement defects of the limbs and die shortly after birth [63]. In these mice, substantial portions of peripheral sensory and sympathetic neurons are absent, while motor neurons are not affected. Moreover, spinal proprioceptive afferents and their peripheral sense organs (muscle spindles and Golgi tendon organs) are completely absent in homozygous NT3 mutant mice [63]. NT4-deficient mice are viable, but exhibit loss of sensory neurons in the nodose-petrosal and geniculate ganglia [234]. Targeted disruption of TrkB, a receptor for BDNF, NT3, and NT4, results in severe nervous system lesions and neonatal death [62]. Knockout of the neurotrophin receptor TrkC in mice results in loss of proprioceptive neurons and early postnatal death [235]. Histological examination of these mice revealed severe cardiac defects such as atrial and ventricular septal as well as valvular defects including pulmonic stenosis. Targeted mutation of the gene encoding the neurotrophin receptor p75NTR leads to deficits in the peripheral sensory nervous system in mice [236].

### 4.2. TGFβ Signaling in the Brain

**Figure 5:**
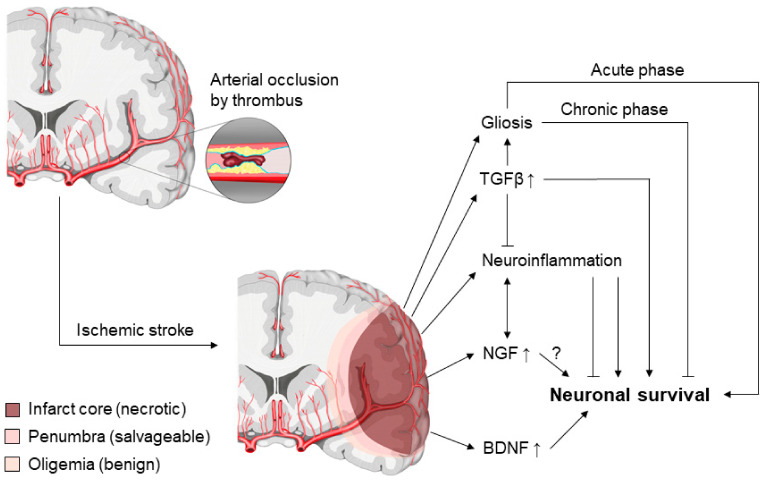
Roles of TGFβ and neurotrophins in recovery from ischemic stroke. Ischemic stroke is induced by a thrombus occluding a brain artery, which impairs blood supply distal to the occlusion. Neurons within the infarct core (<12 mL blood/100 g tissue/min) die rapidly and become necrotic. Neurons within the penumbra (12–22 mL blood/100 g tissue/min) can recover depending on timely reperfusion and the action of different neuroprotective factors such as TGFβ and neurotrophins. Neurons within the oligemia (22–35 mL blood/100 g tissue/min) usually recover completely from the mild ischemia, irrespective of treatment. Gliosis and neuroinflammation are a response to the tissue damage and regulated by TGFβ and neurotrophins. They can be both beneficial and detrimental to post-stroke recovery and neuronal survival. TGFβ = transforming growth factor β, NGF = nerve growth factor; BDNF = brain-derived neurotrophic factor.

During CNS homeostasis in humans, TGFβ ligands are only weakly expressed in microglia and neurons, whereas TGFβ receptors are mainly expressed in microglia, astrocytes, and endothelial cells, albeit at low levels [237]. During CNS inflammation in multiple sclerosis [237] as well as in a rat model of ischemic stroke [15], expression of TGFβ ligands and receptors is upregulated in microglia and astrocytes, a typical hallmark of gliosis [238]. Increased expression of TGFβ1 is also observed in human brain tissue after ischemic stroke (Figure 5) [16]. TGFβ family signaling has been shown to play an essential role in glial and neuronal differentiation, development, and function [239]. Deletion of *Tgfb1* in mice results in a widespread increase in degenerating neurons and prominent microgliosis [240]. Neocortical neurons in mice lacking *Tgfbr2* fail to initiate axons during development [241]. Moreover, TGFβ signaling also critically regulates developmental CNS angiogenesis as well as BBB formation. In fact, TGFβ signaling in endothelial cells, but not neuroepithelial cells, is essential for cerebrovascular development [242]. While mice with endothelial-specific deletion of *Tgfbr2* or *Alk5* (*Alk1^Cre/+^; Tgfbr2^flox/flox^* or *Alk1^Cre/+^; Alk5^flox/flox^*) display impaired forebrain angiogenesis and forebrain hemorrhage starting at E11.5, mice with neuroepithelial-specific deletion of *Tgfbr2* (Nestin-Cre; *Tgfbr2^flox/flox^*) have no apparent embryonic phenotype [242]. The latter finding is consistent with another study demonstrating that ablation of *Tgfbr2* using a different neuroepithelial-specific Cre driver (Emx1-Cre) results in no overt cortical phenotype [243]. In contrast, neuroepithelial-specific deletion of *Tgfbr2* using a *Foxg1^Cre^* knock-in allele (Foxg1^Cre/+^; Tgfbr2^flox/flox^) resulted in reduced vascular branching and an atypical, clustered appearance of blood vessels as well as intracerebral hemorrhage [244]. The authors of the study above speculate that heterozygous deletion of *Foxg1* (*Foxg1^Cre^* is a null allele), in addition to homozygous deletion of *Tgfbr2* in their mouse model, results in the observed phenotype. While the requirement for neuroepithelial TGFβ signaling in CNS angiogenesis is unclear, paracrine activation of latent extracellular TGFβ is mediated by neuroepithelial integrin αvβ8 during development [245,246] and by astrocytic integrin αvβ8 in the adult [247].

### 4.3. A Brief Overview: Ischemic Stroke

Ischemic stroke (ischemic cerebral infarction) is a major cause of mortality and morbidity in humans, as our brain, unlike other organs, has very limited ability to self-repair and regenerate [3]. Ischemic cerebral infarction is usually caused by occlusion of one or more brain arteries by a thrombus or thrombi (Figure 5). Thrombi can originate from in situ arterial thrombosis or from thromboembolism. A frequent cause of arterial thrombosis and embolism is the rupture of an atherosclerotic plaque in a large artery such as the aorta or carotid artery. However, thromboembolisms can also be of cardiac origin with atrial fibrillation, heart valve disease, and heart failure being the most frequent risk factors. Another cause of stroke is small vessel disease, which is associated with elevated blood pressure and diabetes mellitus and is particularly common in the Asian population. A dissection or tear in the intima of an artery and subsequent thrombosis or thromboembolism is a less common cause of stroke, but is most prevalent in younger patients. Currently, the only therapeutic options for ischemic stroke patients are thrombolytic treatment using tissue plasminogen activator (tPA) or endovascular thrombectomy using a catheter. However, the time window for tPA treatment is very narrow: It has to be administered within 4.5 h after the onset of symptoms, after which the detriments outweigh the benefits. Hence, only 15–20% of patients qualify for tPA and/or thrombectomy therapy [3].

Neuronal cell death in the infarct core is the major immediate consequence of stroke and starts within minutes after the onset of ischemia (Figure 5) [248]. However, even once blood supply to the infarcted area is restored, processes such as reperfusion injury, inflammation, and tissue remodeling can lead to secondary neuronal cell death [248]. Intriguingly, evidence from animal experiments suggests that a limited extent of neurogenesis occurs after stroke [249]. Neuronal stem cells (NSC) with the capacity to differentiate into neurons and glial cells usually originate from the subgranular zone in the dentate gyrus of the hippocampus and the subventricular zone [249]. In rodent stroke models, NSC use the rostral migratory stream to move to the ischemic penumbra, the border zone of the infarcted area [249]. NSC then replace injured neurons or glial cells and help with the remodeling and reorganization processes [249]. There is evidence that neurogenesis can also occur in adult human brains after stroke [250], however, adult human neurogenesis is a highly controversial subject [251].

Angiogenesis within the infarcted area of the brain might also help with stroke recovery by augmenting nutrient supply and repair processes and by providing a vascular niche for NSC migration [252]. In human stroke patients, high levels of pro-angiogenic factors in plasma are associated with milder neurologic deficits, whereas high levels of anti-angiogenic factors predict a worse long-term functional outcome [253]. Impaired angiogenesis after stroke is frequently seen in the elderly and in patients with hypertension or diabetes mellitus and is associated with poor functional recovery [252]. However, it is still uncertain whether post-stroke angiogenesis improves neurologic recovery. Brain injuries also trigger an extensive glial response, referred to as reactive gliosis, which is characterized by activation and proliferation of astrocytes and microglial cells accompanied by increased expression of glial fibrillary acidic protein (GFAP) and CD68, respectively [254]. Reactive gliosis eventually leads to the formation of a glial scar replacing the damaged tissue. The glial response to ischemic stroke has been studied in a number of animal models using photothrombosis as well as transient or permanent middle cerebral artery occlusion (t/pMCAO) [255,256,257]. Recent studies show that reactive gliosis exerts both beneficial and detrimental effects on restoring neuronal function after stroke [258]. In the acute phase, reactive gliosis is crucial for sealing the lesion site, remodeling the tissue, and temporally and spatially controlling the local immune response [258]. The glial barrier seals off the infarcted area to maintain extracellular ion and fluid balance, prevent an overwhelming inflammatory response, and scavenge free radicals [258]. Furthermore, the glial scar promotes angiogenesis, increasing the nutritional, trophic, and metabolic support of surviving neurons [258]. On the other hand, the glial scar impedes axon regeneration and inhibits recovery of neuronal function in the chronic phase [258].

### 4.4. Neurotrophins and Ischemic Stroke

The impact of neurotrophins in the context of stroke has been studied extensively. Expression of the neurotrophin BDNF has been shown to be upregulated in particular in the border zone (penumbra) of the infarct indicating a potential role in post-stroke recovery (Figure 5) [13,14]. In fact, heterozygous BDNF-deficient (BDNF^+/−^) or homozygous NT4-deficient (NT4^−/−^) mice display increased susceptibility to transient middle cerebral artery occlusion (tMCAO)-induced ischemic injury [259]. BDNF^+/−^ and NT4^−/−^ mice have 91% and 68% larger cerebral infarcts, respectively, than wild-type mice [259]. Furthermore, enhanced angiogenesis in the ischemic penumbra correlates with increased survival following stroke [260]. Intriguingly, and comparable to MI [98], the Val66Met BDNF polymorphism negatively impacts locomotor functions and the angiogenic response following experimentally induced stroke in rodents [261]. Mice with homozygous knock-in of the human BDNF Val66Met variant (BDNF^Met/Met^) also demonstrate reduced stroke-induced BDNF expression [261]. Moreover, clinical studies show a correlation of the Val66Met *BDNF* polymorphism with poor clinical outcome following hemorrhagic stroke [262,263].

Currently, several promising approaches using neurotrophins to (1) protect neurons from ischemia-reperfusion injury and to (2) mobilize neural stem cells (NSC) are being developed [264,265,266]. A major obstacle in delivering exogenous neurotrophins to the brain is the blood-brain barrier (BBB). To overcome this physiological barrier, anti-transferrin-neurotrophin conjugates as well as neurotrophin-vasoactive intestinal peptide (VIP) fusion proteins, conferring neurotrophins the ability to cross the BBB, are being developed [265].

High serum NGF concentrations are associated with good functional outcome following acute ischemic stroke in humans [267], indicating that BBB-permeable NGF conjugates could be useful therapeutic agents for stroke patients. However, so far, the neurotrophin BDNF has shown the most promising results in pre-clinical studies using rodent stroke models. It has been reported that AMPA receptor-induced local BDNF signaling mediates motor neuron recovery in a mouse model of focal stroke [268]. Furthermore, BDNF administration mediates oligodendrocyte differentiation and myelin formation in a rat model of subcortical ischemic stroke [269]. Endothelial nitric oxide synthase (eNOS) regulates BDNF expression and neurogenesis in the ischemic brain after permanent occlusion of the right middle cerebral artery in mice [270].

NGF and its receptors, TrkA and p75^NTR^, are upregulated in experimentally induced encephalomyelitis in mice [271], a finding that supports other studies suggesting cross-talk between the nervous system and the immune system via NGF [272,273,274,275,276]. Post-ischemic inflammatory responses are known to cause secondary neuronal damage in animal stroke models. Interleukin-1-deficient mice, for example, show ~70% reduction in infarct volume after focal brain ischemia compared to their wild-type littermates [277]. A beneficial effect of administration of a recombinant interleukin-1 receptor antagonist to rats after hypoxia-ischemia-induced cerebral infarction has also been shown [278]. Inhibition of microglia activation by isoflurane or edavarone reduces post-stroke infarct size in rats [279,280]. A circulating antibody against tumor necrosis factor α protects the rat brain from reperfusion injury [281]. However, clinical trials aiming at inhibiting neuroinflammation in stroke patients have been unsuccessful so far [282,283]. In fact, in recent years the role of post-ischemic neuroinflammation has been shown to be more complex and both beneficial and detrimental depending on the animal model, species, and type of elicited immune response (Figure 5) [284,285,286,287].

In conclusion, three lines of evidence show a high therapeutic potential of neurotrophins for the treatment of ischemic stroke in humans: (1) Neurotrophin knock-out mice display increased neuronal cell death and infarct volume after experimentally induced stroke. (2) Several promising preclinical studies show beneficial effects of exogenous neurotrophin administration in rodent stroke models. (3) Human genetics data show a correlation between certain neurotrophin polymorphisms and poor clinical outcome following hemorrhagic stroke.

### 4.5. TGFβ and Ischemic Stroke

Based on its pleiotropic function, it is not surprising that TGFβ (TGFβ1-3) plays an important role in recovery from ischemic stroke [288]. The mechanisms by which TGFβ mediates neuroprotection include anti-inflammatory, -apoptotic, -excitotoxic actions as well as promotion of scar formation, gliosis, angiogenesis, and neuroregeneration [288].

Ischemic stroke-induced BBB breakdown might be one mechanism leading to TGFβ signaling activation. In fact, recently it has been shown that intracerebral injection of serum albumin into rodents (mimicking BBB breakdown), results in activation of TGFβ signaling in astrocytes [289]. It has been reported that the TGFβ1/Smad3 signaling pathway suppresses apoptosis in cerebral ischemic stroke in rats [290]. Furthermore, TGFβ1 protects hippocampal neurons against degeneration caused by transient global ischemia [291] and mediates neuronal rescue after hypoxic-ischemic brain injury [292]. On the other hand, using a rat stroke model, it was suggested that TGFβ1 released by macrophages in the infarct area mediates microglia activation in the peri-infarct area and potential secondary damage to peri-infarct neurons [293]. Overexpression of SMAD3 using a recombinant adeno-associated virus in a rat model of cerebral ischemia/reperfusion mediated neuroprotective effects through anti-inflammatory and anti-apoptotic pathways [294]. Whether these effects were mediated by direct action on post-ischemic neurons or by indirect action on glial cells or both was not addressed. In another study, transgenic mice expressing an astrocyte-specific dominant negative from of TGFβR2 displayed reduced subacute neuroinflammation after experimentally induced stroke [295].

Besides neuroprotection after stroke, TGFβ signaling might also impact the incidence of stroke itself. Cell type-specific deletion of *Tgfbr2* in myeloid cells, i.e., granulocytes and monocytes, in mice results in cerebrovascular inflammation in the absence of significant pathology in other tissues, culminating in stroke and severe neurological deficits, indicating a crucial anti-inflammatory role of TGFβ signaling in myeloid cells within the CNS [296]. In a population-based study (“The Rotterdam Study”) five different *TGFB1* polymorphisms (−800G/A, −509C/T, Leu10Pro, Arg25Pro, and Thr263Ile) were analyzed for correlation with myocardial infarction and stroke. A significantly increased risk of stroke was found for the −509C/T allele as well as the Leu10Pro allele [297]. Interestingly, no association between the *TGFB1* polymorphisms and myocardial infarction was observed. The Leu10Pro polymorphism lies within the signal peptide of TGFβ1 and has been shown to result in a 2.8-fold increase in protein secretion in vitro [298]. In line with the pleiotropic functions of TGFβ, elevated expression levels of this cytokine may be atherogenic and increase the risk of ischemic stroke, but also promote neuroprotection after stroke.

Moreover, mutations in several genes of the TGFβ signaling pathway, e.g., ALK1, ALK5, ENG, SMAD4, and TGFBR1/2, are known to cause Marfan or Loeys-Dietz syndrome, both frequently associated with vascular pathologies such as intracerebral hemorrhages or hemorrhagic hereditary telangiectasia [244,299,300,301,302]. Consistent with its pleiotropic function, TGFβ signaling has both neuroprotective and detrimental roles in brain ischemia. Neuroprotection is mediated by its anti-inflammatory, -apoptotic, and -excitotoxic actions as well as by promoting scar formation, angiogenesis, and neuroregeneration.

On the other hand, elevated expression levels of this growth factor have been associated with increased risk of atherosclerosis and ischemic stroke [297].

### 4.6. TGFβ-Neurotrophin Cross-Talk in the Brain

Since both TGFβ and neurotrophin signaling are upregulated during ischemic stroke and in post-ischemic inflammation, it is tempting to speculate that a cross-talk exists between these two signaling pathways. In fact, an essential role of TGFβ family signaling in glial and neuronal differentiation, development, and function has been well established [239]. In dorsal root ganglia cell cultures, neuronal survival mediated by NGF, NT-3, and NT-4 is TGFβ-dependent [57]. Furthermore, TGFβ induces expression of NGF in cultured astrocytes and subsequent autocrine NGF signaling results in upregulation of neural adhesion molecule L1 [303]. Astrocytic neural adhesion molecule L1 in turn increases neurite outgrowth of co-cultured dorsal root ganglion neurons [303]. Overall, the TGFβ-neurotrophin cross-talk in the brain likely includes TGFβ-mediated upregulation of NGF and potentially other neurotrophins as well as direct enhancement of neurotrophin signaling.

## 5. Conclusions

**Figure 6:**
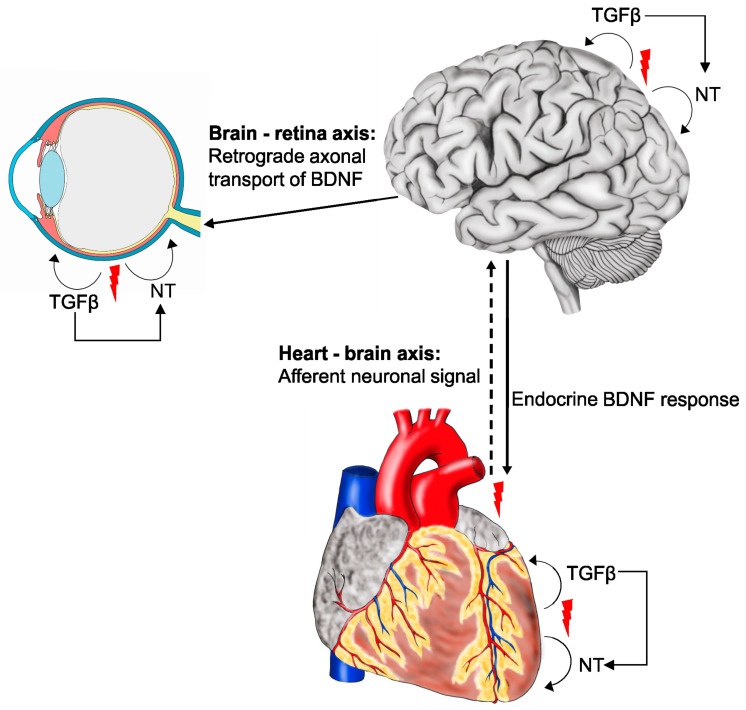
The heart-brain and brain-retina axis. Neurotrophins (NT) and TGFβ are upregulated following ischemic insults (red lightning symbol) to the heart, retina, and brain. Following myocardial infarction, the heart-brain axis is activated by transmitting mechano- and chemo-sensitive information through cardiac afferent fibers (dotted arrow) to the brain, which responds by releasing BDNF to the bloodstream. Thus, BDNF promotes recovery from myocardial infarction by endocrine and paracrine actions. TGFβ directly affects repair processes following ischemic insults. Moreover, it has the ability to increase the expression of NT. Additionally, retrograde transport of neurotrophins is essential for survival of retinal ganglion cells. This close relationship could be defined as a brain-retina axis. TGFβ = transforming growth factor β, NT = neurotrophin, BDNF = brain-derived neurotrophic factor.

Neurotrophin and TGFβ signaling play fundamental roles in development and maintenance, but also in inflammation and repair following ischemic insults or (neo-) vascular pathologies in the heart, the retina, and the brain (Figure 6). Subsequent to myocardial infarction, mechano- and chemo-sensitive information is transmitted through cardiac afferent fibers to the brain, which reacts by releasing BDNF into the blood stream—a scenario that is therefore designated as the ‘heart-brain axis’ (Figure 6). Additionally, retrograde transport of neurotrophins is essential for the survival of retinal ganglion cells. We therefore named this close relationship the ‘brain-retina axis’. While a beneficial role of neurotrophin signaling for tissue protection and repair has clearly been demonstrated, the role of TGFβ signaling in post-insult repair and outcome is more diverse. Given the fact that TGFβ signaling has the potential to regulate neurotrophins and enhance neurotrophin mediated signaling, it is tempting to speculate that these signaling pathways cooperate with the overall aim of protecting the tissue from harmful events. However, impact of TGFβ signaling on inflammation and repair is clearly dependent on the time point of its inhibition and the affected cell type. Consequently, when applying therapeutic modulation of TGFβ signaling the consideration of timing of interference and the targeted cell types are of utmost importance.

## Data Availability

Data sharing not applicable.

## Abbreviations

DR	diabetic retinopathy
AMD	age-related macular degeneration
TGFβ	transforming growth factor β
CNS/PNS	central and peripheral nervous systems,
BDNF	Brain-derived neurotrophic factor
NGF	nerve growth factor
NT-3	neurotrophin-3
NT-4/5	neurotrophin-4/5
TrkA–C	tropomyosin receptor kinase A–C
p75^NTR^	neurotrophin receptor p75
TGFβR1/2	TGFβ receptor types 1 and 2
BMP	bone morphogenetic protein
GDF	growth differentiation factors
ALK1/5	activin A receptor like type 1/5
SMAD	abbreviation refers to “small worm phenotype” (SMA) and “mother against decapentaplegic” (MAD), which are homologs of C. elegans and drosophila
ECM	extracellular matrix
MI	myocardial infraction
BAX	BCL2 Associated X, Apoptosis Regulator
BCL2	BCL2 Apoptosis Regulator
VEGF	vascular endothelial growth factor
CAP	capsaicin
GW788388	small-molecule inhibitor of TGFβR1
BRB	blood-retinal barrier
BBB	blood-brain barrier
RPE	retinal pigment epithelium
RGC	retinal ganglion cells
CNV	choroidal neovascularization
tPA	tissue plasminogen activator
NSC	neuronal stem cells
t/pMCAO	transient or permanent middle cerebral artery occlusion
eNOS	endothelial nitric oxide synthase
ENG	endoglin
